# Influence of maxillary canine torque variations on the perception of smile esthetics among orthodontists and laypersons

**DOI:** 10.1590/2177-6709.24.1.053-061.oar

**Published:** 2019

**Authors:** Thiago Correia Barbosa Lemos, Juliana de Brito Vasconcelos, Bianca Mota dos Santos, Andre Wilson Machado

**Affiliations:** 1 Universidade Tiradentes, Faculdade de Odontologia, Departamento de Ortodontia (Aracaju/SE, Brazil).; 2 Universidade Federal da Bahia, Faculdade de Odontologia, Departamento de Ortodontia (Salvador/BA, Brazil).

**Keywords:** Smile esthetics, Dental esthetics, Orthodontics

## Abstract

**Objective:**

The purpose of this study was to determine the perception of smile esthetics among orthodontists and laypersons with respect to unilateral maxillary canine torque variations in a frontal smile analysis.

**Methods:**

Full face and close-up smile photographs of two subjects (1 man and 1 woman) were used. Both smiles displayed healthy maxillary anterior dentitions. The images were digitally altered to obtain a bilateral 0° torque in the maxillary canines. From this image, unilateral variations of the left canine were made with -15°, -10°, -5°, 0°, +5°, +10° and +15°. Final images were randomly assembled into an album that was given to 53 orthodontists and 53 laypersons. Each rater was asked to evaluate the attractiveness of the images using visual analog scales. Data collected were statistically analyzed with one-way analysis of variance with Tukey *post-hoc* test and the unpaired Student *t* test.

**Results:**

For orthodontists, most attractive smiles were those with 0°, -5° and -10°. For laypersons, most attractive smiles were those with 0°, -5°, -10°, -15° and + 5°. For both groups, the lowest scores were given for the smiles with +10° and +15° torque. When comparing the perceptions of the orthodontists and laypersons, they did not show statistical differences in most situations. Moreover, in general, there was no significant difference between the full-face and close-up assessments of the smiles.

**Conclusions:**

The present findings indicated that smiles with unilateral palatal (negative values) maxillary canine torque variations were more tolerated than smiles with buccal crown torque (positive values) variations.

## RESUMO

**Objetivo:**

o objetivo do presente estudo foi avaliar a percepção estética do sorriso, por ortodontistas e leigos, em relação a variações unilaterais do torque do canino superior, em uma análise frontal do sorriso.

**Métodos:**

foram utilizadas fotografias de face total e de sorriso aproximado de dois indivíduos (1 homem e 1 mulher). Ambos os sorrisos exibiam dentição anterossuperior saudável. As imagens foram alteradas digitalmente para obtenção de um torque bilateral de 0° nos caninos superiores. A partir dessa imagem, variações unilaterais do canino esquerdo foram realizadas com -15°, -10°, -5°, 0°, +5°, +10° e +15°. As imagens finais foram montadas aleatoriamente em um álbum que foi entregue a 53 ortodontistas e 53 leigos. Cada avaliador foi orientado a avaliar a atratividade das imagens com escalas visuais analógicas. Os dados coletados foram analisados estatisticamente com análise de variância ANOVA com pós-teste de Tukey e teste *t* de Student não pareado.

**Resultados:**

para ortodontistas, os sorrisos mais atraentes foram aqueles com 0°, -5° e -10°. Para os leigos, os sorrisos mais atraentes foram aqueles com 0°, -5°, -10°, -15° e +5°. Para ambos os grupos, as pontuações mais baixas foram dadas para os sorrisos com torque de +10° e +15°. Ao comparar as percepções dos ortodontistas e leigos, não foram encontradas diferenças estatísticas na maioria das situações. Além disso, em geral, não houve diferença significativa entre as avaliações de face total e de sorriso aproximado.

**Conclusões:**

esses achados indicam que os sorrisos com variações unilaterais no torque do canino superior para palatino (valores negativos) foram mais tolerados do que os sorrisos com variações no torque da coroa para vestibular (valores positivos).

## INTRODUCTION

Orthodontics is going through a transition process over the years related to its therapeutic goals. With the growing increase in demand for an attractive smile, the orthodontist needs to add parameters of esthetic smile ideals to their treatment goals, in order to meet the patients’ needs.^1^ ^,^ ^2^

The definition of an attractive smile is based on widely studied aspects such as symmetry and proportion between the maxillary central incisors, adequate gingival exposure, convex smile arch, moderate buccal corridors and proper design of the gingival margins in the esthetic zone.^3^ ^-^ ^6^ However, few studies investigated the role of the maxillary canines on the smile’s attractiveness. These teeth play a prominent role in the dental arch due to its location in a transition zone between the anterior and posterior teeth.^7^

In this area, the presence of asymmetries on the incisal edges^8^ and gingival margins have already been analyzed.^4^ According to Pinho et al^8^, when investigating the esthetic perception of maxillary canines incisal edge asymmetries, they found that the discrepancies of up to 2 mm were not detected by orthodontists, prosthodontists and laypeople. According to Correa et al.,^4^ when investigating the esthetic influence of the presence of asymmetries between the gingival margins of the maxillary canines, orthodontists did not perceive the asymmetries between the maxillary canine gingival margins up to 0.5 mm, and laypeople did not perceive asymmetries up to 1.0 - 1.5 mm.

However, the influence of unilateral maxillary canines torque variations on smile esthetics perception has not yet been evaluated. It is hypothesized that variations in this aspect may influence in the esthetic perception by interfering in the harmonic ratio of the maxillary teeth in a frontal view.^5^

Considering the wide variety of canines torque,^2^ ^,^ ^9^ ^,^ ^10^ either by anatomical features or the various pre-adjusted orthodontic appliance prescriptions^5^, some questions can be raised: If an asymmetry is related to the maxillary canine torque, what do laypeople and orthodontists perceive? In other words, what is the threshold for these people when evaluating unilateral canine torque variations? Would be the correction of minor maxillary canine torque variations an overtreatment more than an esthetic need?

Therefore, the aim of this study was to determine the perception of smile esthetics among orthodontists and laypeople with respect to the presence of unilateral maxillary canine torque variations in a frontal smile analysis (full-face and close-up views). The null hypothesis tested was that the presence of these torque variations is equally rated as attractive by orthodontists and laypeople.

## MATERIAL AND METHODS

This research was evaluated and approved by the Research Ethics Committee of the Dental School from the Tiradentes University protocol no. 1.740.448, and registered by National Research Ethics Committee protocol no. 57689315.3.0000.5371. All participants signed an informed consent form. 

Questionnaire data from a pilot study with ten subjects in each group were used in the sample size calculation. Based on the level of significance (alpha) of 0.01 and the effect size of 0.90, the sample size was calculated to achieve 80% power. This calculation showed that 53 subjects in each group were necessary.

Twenty-eight images (14 photographs of full-face view and 14 close-up views of the smile) were used from 2 subjects (1 man and 1 woman), who were volunteer patients (age between 25 and 30 years), with attractive smiles and no apparent facial asymmetry. The selected images were published elsewhere and were chosen because they fulfill some principles of an attractive smile.^4^

Photographs were digitally altered using Adobe Photoshop software (CS3, Adobe Systems, San Jose, California) to produce symmetrical images and were then retouched to adjust brightness and contrast.^2^ ^-^ ^4^ ^,^ ^11^ ^-^ ^13^ The image was then condensed to achieve an image with measurements identical to those on the actual patient (1:1 magnification ratio). In order to do this, the right upper central incisor width, measured in the patient, was used as a reference.^2^ ^-^ ^4^ ^,^ ^11^ ^,^ ^12^

**Figure 1:**
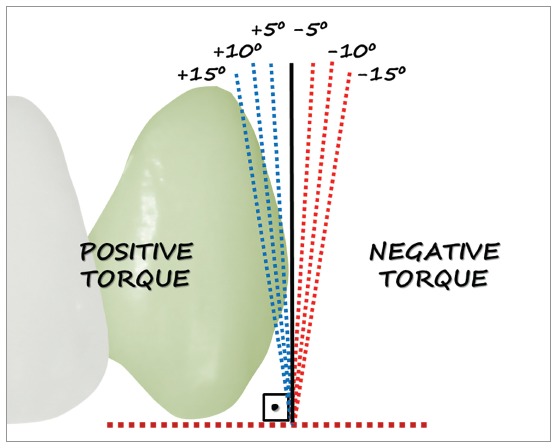
Representation of changes of 5^o^, 10^o^ and 15^o^ in the torque of the left canine, on the buccal direction (positive torque) and lingual (negative torque).

For each image a change was made in the right canine torque in order to obtain a neutral torque (0^o^), which served as control during the study. In order to do this, two references were considered: a horizontal line connecting the right and left canines tips and a line tangent to the buccal surface of the canine crown. The former line was drawn at the height of contour level of the crown’s most disto-buccal aspect. The right canine (0^o^) served as a reference for the torque variations on the left side. Alterations were measured in degrees, in 5^o^ increments (5o, 10o and 15o) of the left canine in relation to the right side (Fig 1). Alterations in the buccal direction were labeled as positive and in the palatal direction, as negative.

**Figure 2:**
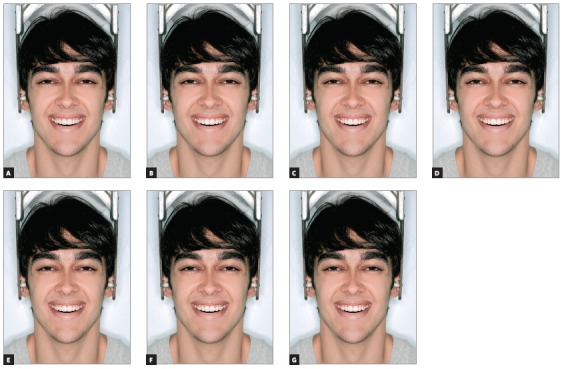
Full face views of man, with asymmetry of the upper left canine torque ranging in 5^o^: A) -15^o^; B) -10^o^; C) -5^o^; D) control (0^o^); E) +5^o^; F) +10^o^; G) +15^o^.

**Figure 3:**
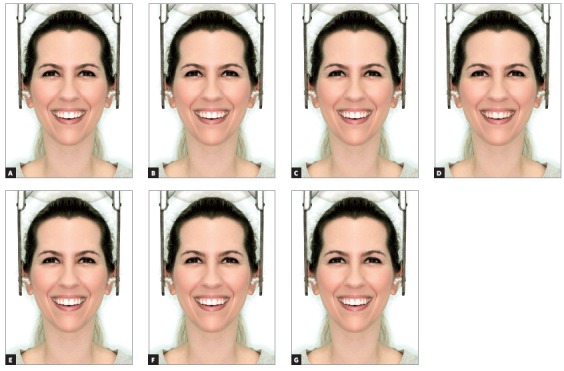
Full face views of woman, with asymmetry of the upper left canine torque ranging in 5^o^: A) -15^o^; B) -10^o^; C) -5^o^; D) control (0^o^); E) +5^o^; F) +10^o^; G) +15^o^.

**Figure 4:**
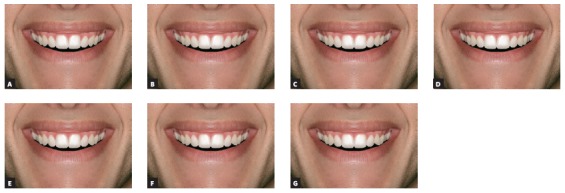
Close-up views of man, with asymmetry of the upper left canine torque ranging in 5^o^: A) -15^o^; B) -10^o^; C) -5^o^; D) control (0^o^); E) +5^o^; F) +10^o^; G) +15^o^.

**Figure 5:**
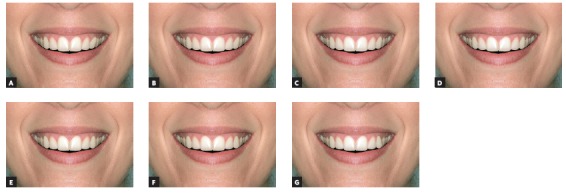
Close-up views of woman, with asymmetry of the upper left canine torque ranging in 5^o^: A) -15^o^; B) -10^o^; C) -5^o^; D) control (0^o^); E) +5^o^; F) +10^o^; G) +15^o^.

After these alterations, 14 images were obtained, 7 of full-face (Figs 2 and 3) and 7 of the close-up smile (Figs 4 and 5), all related to changes made in the left canine torque (-15^o^, -10^o^, -5^o^, 0^o^, + 5^o^, + 10^o^ and + 15^o^).

Final images were printed on A3 size (29.7 cm x 42.0 cm) and a photographic album was compiled with all images randomly arranged and coded using numbers and letters.^2^ ^-^ ^4^

The album was given to 106 evaluators, 53 orthodontists (28 male and 25 female, with mean ages of 34.14 years and 33.36 years, respectively) and 53 laypersons (23 male and 30 female, with mean ages of 35.80 years and 33.33 years, respectively) with a college education but no dental background. Before each evaluation, the evaluator members received information about the study and each one rated the attractiveness of the photographs on a form with visual analog scales (one for each image), anchored by word descriptors at each end: “very unattractive“ at the left and “very attractive” at the right. All evaluators marked a point along the scale according to their perception of smile esthetics. The scores were then measured in millimeters, with a digital caliper (Starrett, Suzhou, China).^2^ ^-^ ^4^ ^,^ ^8^ ^,^ ^10^ ^-^ ^12^

In order to assess the reliability of the method, fifteen raters (30% of each group) from each group were randomly selected.^2^ ^-^ ^4^ ^,^ ^11^ ^,^ ^14^ They were asked to evaluate two series of images (one containing a full-face view and the other with the close-up view) of the album in which there were two identical images. Intraclass correlation coefficients (ICC) were used to compare the scores for those images, in order to determine intra-rater agreement. High levels of reliability were found, since all coefficients were greater than or equal to 0.77 for both groups of raters.

Descriptive statistics were presented as means and standard deviations. Differences in mean scores of 7 levels of unilateral torque were evaluated using one-way analysis of variance (ANOVA) with the Tukey *post-hoc* test. In order to compare the distributions of mean scores between the images of full-face and close-up views and also among orthodontists and laypeople, Student’s *t* test was used. The level of significance was established at 5%.

## RESULTS

When comparing the full-face and the close-up views, in most cases (12 out of 14 ratings) there were no statistical differences between these two views (Tables 1 and 2). After this result, the close-up smile view was used as the reference to address the results.

**Table 1:** Mean and standard deviations of VAS scores of the attractiveness of full-face and close-up smile of the man with unilateral variation of canine torque for both groups (p > 0.05).

Variable	Full face		Close-up		P
	Mean	SD	Mean	SD	
-15°	55.8	17.3	53.9	18.8	0.216
-10°	59.5	16.6	58.2	19.3	0.293
-5°	63.8	20.5	59.7	18.9	0.067
0°	63.3	17.1	62.6	18.4	0.387
+5°	55.7	18.7	52.7	19.7	0.127
+10°	49.7	19.2	42.0	20.2	0.002
+15°	43.1	22.3	37.5	19.5	0.081

**Table 2:** Mean and standard deviations of VAS scores of the attractiveness of full-face and close-up smile of the woman with unilateral variation of canine torque for both groups (p > 0.05).

Variable	Full face		Close-up		P
	Mean	SD	Mean	SD	
-15°	48.1	20.1	55.5	20.8	0.005
-10°	55.9	22.1	56.3	19.9	0.440
-5°	63.9	18.5	61.4	20.2	0.172
0°	59.4	21.1	61.1	21.0	0.277
+5°	59.1	21.4	57.7	19.9	0.311
+10°	47.3	21.1	45.6	19.7	0.270
+15°	43.4	20.4	44.6	21.7	0.345

Table 3 shows the results for the close-up smile of the man. According to the orthodontists, the highest scores were attributed to the smiles with unaltered torque (0°) and for those with -5° and -10° torque (*p*< 0.05). They also appointed the lowest scores for the smiles with +10° and +15° torque (*p*< 0.05). To laypeople, the only smiles considered less attractive were those with +10° and +15° torque (*p*< 0.05).

**Table 3:** Means and standard deviations of VAS scores of the attractiveness of close-up smile of the man with unilateral variation of canine torque (p < 0.05).

Variable	Orthodontist			Laypeople			Ortho. X Lay.
	Mean	SD	Result*	Mean	SD	Result*	
-15°	53,48	14,64	B,C	54,37	22,45	A,B	
-10°	57,29	16,23	A,B	59,16	22,21	A,B	
-5°	62,15	15,21	A	57,41	21,93	A,B	
0°	61,99	14,77	A,B	63,35	21,65	A	
+5°	48,89	14,77	C	56,54	23,30	A,B	
+10°	36,28	14,68	D	47,84	23,28	B,C	†
+15°	33,19	14,71	D	41,92	22,68	C	†

* Variables with the same letter are not statistically different (p < 0.01); † statistical differences between groups of examiners (p < 0.01).

Table 4 shows the results for the close-up smile of the woman. For the orthodontists group, the most attractive smiles were those with unilateral torque of 0°, -5°, -10° and + 5°, while the less attractive were those with +10° and +15° torque (*p*< 0.05). In the assessment of the laypeople, the only smile considered less attractive was the one with +15° torque (*p*< 0.05).

**Table 4:** Means and standard deviations of VAS scores of the attractiveness of close-up smile of the woman with unilateral variation of canine torque (p < 0.05).

Variable	Orthodontist			Laypeople			Ortho. X Lay.
	Mean	SD	Result*	Mean	SD	Result*	
-15°	52.48	20.56	B,C	58.55	20.81	A,B	
-10°	54.98	17.88	A,B	57.80	21.96	A,B	
-5°	62.93	17.54	A	60.00	22.78	A,B	
0°	60.48	17.45	A,B	61.90	24.21	A	
+5°	56.20	17.66	A,B	59.29	21.98	A,B	
+10°	42.89	14.03	C,D	52.19	23.94	A,B	†
+15°	37.08	13.59	D	48.42	25.57	B	†

* Variables with the same letter are not statistically different (p < 0.05); † statistical differences between groups of examiners (p < 0.01).

In general, when comparing the opinions of orthodontists and laypeople, it was observed that there were statistically significant differences in the smiles considered less attractive (+10° and +15°), with higher scores given by the laypeople (*p*< 0.01).

## DISCUSSION

The growing demand of patients looking for an attractive smile in dental offices promoted the inclusion of various esthetic guidelines on treatment goals.^2^ ^,^ ^6^ ^,^ ^7^ Although several aspects of an ideal smile have been published, the role of maxillary canines has not been well established. Given the esthetic importance of these teeth, due to its position in a transition region between the anterior and posterior segment and also due to its leading role for being located at the ends of the esthetic zone, its relationship with the attractiveness of the smile became the objective of this study.^7^

This study digitally created seven smiles with unilateral modifications of the maxillary canine torque from -15° to +15°, in 5° increments. The ppresent results showed that this characteristic partially influenced the smile esthetics perception, which corroborates the findings of Xu et al.^13^ In general, orthodontists and laypeople could not detect torque variations of -10^o^ to 0^o^ and of -15° to +5°, respectively. In other words, orthodontist judged three smiles (0^o^, -5^o^ and -10^o^) and laypeople five smiles (0^o^, -15^o^, -10^o^, -5^o^ and +5^o^) as equally attractive. After carefully evaluating the above information, it is evident that small unilateral maxillary canine torque variations did not influence the smile esthetic perception. With this in mind, when deciding upon the ideal maxillary canine torque of the maxillary canine in a given case, the clinician should give more priority to proper function of these teeth instead of smile esthetics. 

Only one study^13^ was found in the literature evaluating the influence of maxillary canine torque variations on smile esthetics. The authors^13^ bilaterally assessed canine, first and second premolars torque variations. However, other unilateral alterations such as incisal wear^8^ and gingival margins alterations^4^ were well addressed in the literature. Pinho et al.^8^ studied the influence of unilateral canine incisal wear on smile esthetic perception, and found that neither orthodontists nor laypeople were capable of detecting a 2.0-mm asymmetry between right and left sides. Correa et al.^4^ followed the same trend, evaluating the influence of maxillary canines gingival margins asymmetries, and found that orthodontist and laypeople could not detect 0.5-mm and 1.0 to 1.5-mm asymmetry, respectively. The above information shows that unilateral maxillary canines small asymmetries may not be judged as an unattractive characteristic especially for laypeople, which corroborates the present findings. However, it is of paramount importance to highlight that the presence of tooth incisal asymmetries between maxillary central incisors^3^ ^,^ ^14^ is not well tolerated as those unilateral torque asymmetries in the canine area. This finding corroborates a clinical assumption that the closer to the midline, the greater the need of symmetry, and the further away from the midline, gentle asymmetries are more acceptable.^3^ ^,^ ^14^

In a clinical standpoint, maxillary unilateral torque variations are mainly caused by tooth anatomy and morphology variations, tooth wear or abrasion, presence of composite restorations or porcelain veneers and orthodontic tooth movement. With this in mind, the decision-making process to correct maxillary canine torque needs to address these aspects and accomplish an ideal esthetic result. Among all treatment strategies to correct canine torque alterations, orthodontic treatment plays a vital role with its ability to individually apply torque in the bracket-wire configuration. Although these treatment strategies are well documented in the literature, from an esthetic standpoint, an intriguing question can be asked: is it necessary to correct a small maxillary canine torque variation? Otherwise stated, if laypeople cannot recognize a unilateral canine toque variation as unattractive, why should dental specialists need to treat it? 

On the other hand, an important aspect related to orthodontic treatment and maxillary canine torque needs to be carefully addressed. Our findings indicated that smiles with maxillary canine palatal torque (negative values) variations were more tolerated than smiles with buccal crown torque (positive values) variations, which corroborates the findings of Xu et al.^13^ Otherwise stated, laypeople judged as unattractive +10^o^ and +15^o^ torque smiles, whereas all palatal torque situations (-15^o^, -10^o^, -5^o^) were not perceived as unattractive. Our hypothesis to explain the esthetic preference for canine palatal crown torque is related to the golden ratio, which is related to the apparent widths of the maxillary teeth from a frontal view.^6^ ^,^ ^7^ When maxillary canines are buccally torqued (+5^o^, +10^o^, +15^o^), these tooth occupy the apparent width (in a frontal view) of the premolar, and thus, creating an inharmonious smile. In other words, when canines were torqued buccally, first premolar crown was less apparent. 

For this reason, the clinician should include this information in the decision-making process before utilizing mechanics to tip the maxillary canines buccally, such as dental expansion and step-up bends. Another clinical situation that may create an important maxillary unilateral torque variation is the agenesis of maxillary lateral incisors associated with canine substitution.^14^ ^,^ ^15^ In these situations, the canine replaces the missing lateral incisor and the premolar assumes the role of the canine.^4^ It is clear that if the canine substitution is performed unilaterally, a torque asymmetry will be present and thus, we suggest that if the variation is within the esthetic threshold, a clinical procedure such as dental restorations or enamel reshaping might not be necessary.

Since the present article addressed the influence of unilateral maxillary canine torque variations, it is evident that the present findings can not be extrapolated to clinical cases that present bilateral torque alterations. This issue will be discussed in a future article. However, literature^15^ shows that asymmetric alterations make teeth more unesthetic than symmetric alterations.

In this study, we surveyed orthodontists and laypeople. In the literature there is a tendency of these rater groups to have different perceptions, with orthodontists being more rigid.^2.3,15^ In contrast, we found that in only a few situations, orthodontists were more critical in their assessments, specifically in unattractive smiles (+10^o^ and +15^o^, and +5^o^ for the smile of the man). This fact disagrees with other studies that show differences in all evaluations.^2^ ^,^ ^3^

When evaluating the full-face view compared with the close-up view of the smile, no significant difference (*p*> 0.05) was found. These findings agree with other studies^1,4,12^ and suggest that during the assessment of the smile the presence of components of the face - such as hair, nose, eyes and skin tone - did not influence on the perception of smile esthetics for both groups of raters. 

The results of this study were based on averages and should be analyzed with caution due to the following factors: canine torque variations were digitally created and, thus, might not reflect with perfection a clinical situation; images from two patients were used; evaluations were based on the opinions of two specific groups of raters; there is some difficulty in distinguishing this information to a patient due to smile subjectivity.^2^ ^-^ ^4^ ^,^ ^15^ Thus, it is advisable to all clinicians to discuss these results with patients presenting unilateral maxillary canine torque variations, since if there is no functional and/or pathological interference, it can be decided together about the correction or not of this alteration.

## CONCLUSION

According to the results of this study, it can be concluded that:

1. In general, the most attractive smiles for orthodontists were the control (no unilateral torque variation) and the -5° and -10° palatal crown torque. For the laypeople, the most attractive smiles were the control, the -5°, -10°, -15° and +5°. For both groups, the lowest scores were assigned to the smiles with unilateral buccal crown torque of +10° and +15° (*p*< 0.05).
2. In most situations, there were no statistical differences between orthodontists and laypeople. In the assessment of the smiles with +10° and +15° unilateral torque, the orthodontists were more critical in their evaluations, giving lower scores than the laypeople (*p*< 0.01).
3. In most situations, no statistically significant difference (*p*> 0.05) was found between the full-face and close-up assessments of the smiles.
